# Live‐attenuated vaccines in a cryopyrin‐associated periodic syndrome patient receiving canakinumab treatment during infancy

**DOI:** 10.1002/ccr3.1149

**Published:** 2017-09-12

**Authors:** Misa Watanabe, Ryuta Nishikomori, Yuki Fujimaki, Toshio Heike, Akira Ohara, Tsutomu Saji

**Affiliations:** ^1^ Department of Pediatrics Toho University School of Medicine Ota‐ku Tokyo Japan; ^2^ Department of Pediatrics Graduate School of Medicine Kyoto University Kyoto Japan; ^3^ Advanced and Integrated Cardiovascular Research Course in the Young and Adolescence Tokyo Japan

**Keywords:** Canakinumab, cryopyrin‐associated periodic syndrome, infant, live‐attenuated vaccine, neonatal‐onset multisystem inflammatory disease

## Abstract

We successfully immunized the neonatal‐onset multisystem inflammatory disease (NOMID) patient with live‐attenuated vaccines for measles, rubella, varicella, and mumps and achieved sufficient antibody titer under canakinumab therapy without complications.

## Introduction

Cryopyrin‐associated periodic syndrome (CAPS) is a periodic fever syndrome classified as an autoinflammatory disease 1, 2, 3. CAPS is caused by abnormalities in the NLRP3 protein in which a genetic mutation in the NACHT domain causes continuous overproduction of interleukin (IL)‐1*β*, resulting in chronic inflammation and progressive tissue damage 4, 5, 6, 7. Canakinumab is a fully humanized anti‐IL‐1*β* monoclonal antibody that has been successfully used to manage CAPS 8, 9, 10. However, clinical trials of canakinumab have been carried out in patients aged 2 years or older 11. Furthermore, while the efficacy and safety of canakinumab have been recently reported 8, 9, 10, 11, 12, 13, 14, 15, few reports have described administration of this drug in infants 16, 17. Despite the lack of studies, data have highlighted the importance of beginning early treatment in infancy to prevent irreversible organ damage and lifelong disabilities in patients with CAPS.

Inoculation of live‐attenuated vaccines is contraindicated in patients treated with biologic agents. Therefore, live‐attenuated vaccines should be administered before initiating the treatment. However, neither live‐attenuated vaccines nor biologics are recommended in early infancy.

Herein, we present the case of a 4‐month‐old girl with CAPS who exhibited neonatal‐onset multisystem inflammatory disease/chronic infantile neurologic, cutaneous, and articular (NOMID/CINCA) syndrome. In this case, we successfully treated the patient with canakinumab starting at 6 months of age and immunized the patient with live‐attenuated vaccines for measles, rubella, varicella, and mumps, without the occurrence of adverse events.

## Case Presentation

We acquired approval for this case study from the ethics committee of Toho University Hospital (no. 25‐1, 26‐148, and 27‐137). Written informed consent was obtained from the guardians of the patient for publication of this case report and any accompanying images.

The 4‐month‐old girl was admitted to our hospital because of a generalized urticarial rash. She was born via spontaneous vaginal delivery at 38 weeks of gestation to a healthy 39‐year‐old mother after an uncomplicated pregnancy. At birth, her weight was 2340 g (−1.5 SD) and her length was 46 cm (−1.1 SD). The rash appeared immediately after birth and worsened 4 months later; thus, the patient was referred to our hospital.

On the first visit to our outpatient clinic, the patient showed a high inflammatory response upon laboratory blood examination, although she was afebrile. One day before admission for further examination, the patient's temperature rose to 38°C and a daily fever recurred thereafter. There was no family history of skin rash, periodic fever, or other autoinflammatory diseases.

Physical examination showed an urticarial rash measuring 10–20 mm in diameter surrounded by erythema generally distributed over the skin. Audiography assessments did not show any abnormalities, with no joint swelling; however, hematological and serum biochemical examinations revealed a white blood cell count of 17,300/*μ*L, with a differential profile of 5.5% band neutrophils, 30.5% segmented neutrophils, and 61% lymphocytes; C‐reactive protein (CRP) was 59 mg/L; erythrocyte sedimentation rate (ESR) was 38 mm/h; serum amyloid A (SAA) was 251 *μ*g/mL (control ≤ 8); serum IL‐6 was 67.6 pg/mL (control ≤ 4); serum immunoglobulin (Ig) G was 1.127 mg/dL; IgA was 25 mg/dL; and IgM was 126 mg/dL. Tests for autoantibodies were negative. A virological test showed a positive cytomegalovirus (CMV) IgM titer of 3.82 (control < 0.8) and a positive CMV IgG titer of 30.9 (control < 2.0), whereas CMV‐C7HRP and CMV antigenemia were negative; these data indicated a recent CMV infection. The skin biopsy for histopathological examination showed no eosinophilic infiltration, with mild perivascular neutrophil infiltration.

After hospitalization, periodic fevers, aseptic meningitis, and a skin biopsy provided strong indicators of an autoinflammatory disease. An informed consent was obtained from her parents, and we performed genetic testing, which showed a de novo heterozygous mutation in the *NLRP3* gene (c.2263G > A, p.Gly755Arg heterozygous); this mutation has been reported as a CAPS disease‐causing mutation 18.

Accordingly, we started the patient on canakinumab at 6 months old after acquiring approval from the institutional ethics committee (no. 25‐1) of our hospital. A positive clinical response was noted, with complete disappearance of the skin rash and periodic fevers 8.5 h after the first injection (2 mg/kg). The patient met the clinical and serological remission criteria used in the Japanese clinical studies 11, and canakinumab was administered as a single 2 mg/kg dose once every 8 weeks via subcutaneous injection.

## Outcome and Follow‐Up

**Figure 1:**
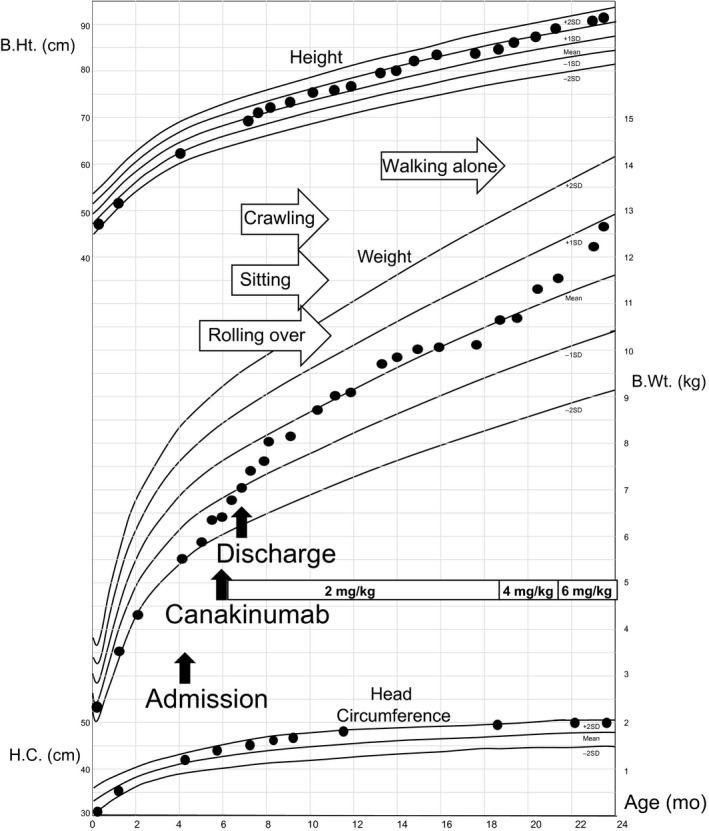
Growth and development of the patient. The patient exhibited normal growth and development after the initiation of canakinumab treatment, with no signs of mental retardation. Mean and standard deviations of height, weight, and head circumference are shown for 0–27 months of age. B.Ht., B.Wt, and H.C. indicate body height, body weight, and head circumference, respectively.

Thereafter, the patient's body weight rapidly increased and motor development progressed normally (Fig. 1). She began to walk at 15 months old and demonstrated normal psychomotor development.

**Figure 2:**
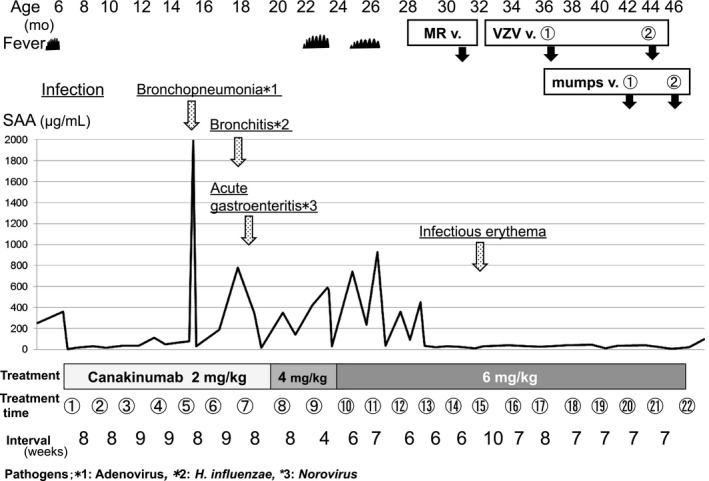
Clinical course and laboratory findings after the initiation of canakinumab therapy. The figure shows the serum amyloid A (SAA) profile during the canakinumab treatment period. The disease‐causing pathogens and diseases are underlined. Inoculation with live‐attenuated vaccines (MR v., VZV v., and mumps v. are measles–rubella vaccine, varicella zoster vaccine, and mumps vaccine, respectively) are indicated by solid arrows.

We detected the presence of *Streptococcus pneumoniae* and *Branhamella catarrhalis* at 13 months old and *Haemophilus influenzae type B* at 15 months old using a suction sputum culture. These pathogens occasionally cause acute bronchopneumonia and acute bronchitis; however, they did not cause severe infection in this patient. In contrast, adenovirus‐induced acute bronchopneumonia and norovirus‐induced gastroenteritis resulted in the patient requiring intravenous treatment for several days (Fig. 2).

Nevertheless, the patient experienced periodic fevers without rash starting at 20 months old, with an elevated CRP of 32 mg/L and SAA of 349 *μ*g/mL. After diagnosing her relapse, we increased the therapeutic dose of canakinumab in a stepwise manner to 6 mg/kg within 6–7 weeks (Fig. 2). The patient is now 47 months old and in clinical and serological remission.

Because viral infections may have triggered or exacerbated the CAPS symptoms, we implemented a normal vaccine schedule; the patient was vaccinated with *Bacillus* Calmette–Guérin (BCG), *Haemophilus influenzae* type B (Hib), 13‐pneumococcal polysaccharide vaccine (PCV13), and diphtheria–pertussis–tetanus‐inactivated polio vaccine (DPT‐IPV) before initiating treatment (Table 1). Additionally, we administered the seasonal influenza vaccine and palivizumab for respiratory syncytial virus (RSV) infection. We then proposed a clinical research project to immunize the patient with live‐attenuated vaccines while under canakinumab treatment, which was approved by the ethics committee of our hospital (no. 26‐148, 27‐137). Thus, the patient received the measles–rubella (MR) vaccine, primary/booster varicella zoster virus (VZV) vaccines, and mumps vaccine 3–4 weeks after the last injection and 4 weeks before the next injection of canakinumab at 31–46 months of age. She had no vaccine‐related adverse effects and achieved sufficient antibody titers (measles IgG titer of ≥128 [control < 2.0]; rubella IgG titer of 31.5 [control < 2.0]; varicella zoster IgG titer of 6.0 [primary], 26.6 [booster; control < 2.0]; and mumps IgG titer of 7.8 [primary], 48.0 [booster; control < 2.0]; Table 1).

**Table 1:** Immunization records and antibody titers after vaccinations

Before treatment					After treatment				
Age (months)	Vaccine	Frequency		Antibody titer	Age (months)	Vaccine	Frequency		Antibody titer
2–4	Hib	3			12	Hib	1		
2–4	PCV13	3			12	PCV13	1		
3–4	DPT‐IPV	3	Pertussis FHA	27	17	DPT‐IPV	1	Pertussis FHA	29
4	**BCG**	1			31	**MR**	1	Measles	≥128
								Rubella	31.5
					37	**VZV①**	1	VZV	6
					44	**VZV②**	Booster	VZV	26.6
					42	**Mumps①**	1	Mumps	7.8
					46	**Mumps②**	Booster	Mumps	48.0
					12–37	Flu	6		

Hib, *Haemophilus influenzae* type B; PCV13, pneumococcus; DPT‐IPV, diphtheria–pertussis–tetanus‐inactivated polio vaccine; FHA, filamentous hemagglutinin; MR, measles–rubella; VZV, varicella zoster virus; BCG, *Bacillus* Calmette–Guérin.

IgG antibody titers of measles, rubella, and varicella viruses were measured by enzyme‐linked immunosorbent assay (ELISA) (Control < 2.0).

Filamentous hemagglutinin (FHA) in *Bordetella pertussis* IgG antibody titers was measured by ELISA (Control < 10.0 EU/mL).

The names in bold type indicate live vaccines.

## Discussion

NOMID/CINCA syndrome is the most severe CAPS phenotype 12, exhibiting major symptoms such as chronic aseptic meningitis, developmental delays, mental retardation, osteoarthropathy, sensorineural hearing loss, and optic neuritis with loss of vision if left untreated 13. We herein report a case of NOMID syndrome that was effectively and safely treated with canakinumab starting at 6 months of age without severe complications. In addition, we successfully immunized the patient with live‐attenuated vaccines.

Because canakinumab is not approved for use in infants younger than 2 years, we initiated treatment after acquiring approval from the institutional ethical committee of our hospital and the patient's parents. We also found three reports of infants treated with canakinumab; however, these cases had not been described in the literature at the initial presentation of our case 13, 16, 17. Although the treatment in these three cases appeared to be effective with no particular or severe adverse effects, higher dosages of canakinumab were needed to control the disease 9, 10, 12. Moreover, these reports highlighted the importance of beginning treatment early in infancy to prevent irreversible organ damage and lifelong disability. A minimum dose of canakinumab was maintained for remission in the present case until 20 months of age. The treatment was entirely effective and safe.

Treatment of genetic disorders, such as CAPS, at infancy may result in complications with vaccinations. However, nonlive vaccines are generally safe and effective in pediatric patients with autoimmune and rheumatic diseases receiving corticosteroids, immunosuppressants, and biologics 19, 20, 21, 22. Recently, the safety and efficacy of live‐attenuated vaccines in rheumatic and autoimmune diseases have also been reported 23, 24, 25, 26. However, these findings are not sufficient to support the safety and immunogenicity of live‐attenuated vaccines in pediatric patients with autoimmune and rheumatic diseases. Thus, live‐attenuated vaccines should be administered to these patients only after acquiring approval from the appropriate institutional review board.

Autoinflammatory diseases are rare, making it difficult to perform studies with large numbers of patients. To date, only seven patients have been described as receiving live‐attenuated vaccines along with antitumor necrosis factor alpha (TNF‐*α*) treatment; no reports have described the use of live‐attenuated vaccines in patients administered anakinra and rilonacept. Five patients received the measles–mumps–rubella (MMR) vaccine, and two patients received the VZV vaccine without severe adverse events. Moreover, very few cases of patients receiving canakinumab therapy and the MMR vaccine have been reported to date; indeed, only one case in which vaccines were accidentally administered is currently described in the literature 9.

Vaccine schedules are different for each country. In Japan, an MR booster and varicella vaccine were included in the national vaccine program in 2006 and 2014, respectively. However, as the mumps vaccine is still optional, it is possible that our patient will be exposed to chickenpox and mumps while in school. Further, it is likely that viral infections exacerbated the initial CAPS symptoms, resulting in delayed treatment. In addition, our hospital is near an international airport. Thus, the patient was at risk of contact with individuals having infectious diseases acquired abroad; several measles outbreaks have occurred in recent years. Considering the risk of contracting vaccine‐preventable diseases and the report of successful MMR immunization of a CAPS patient on canakinumab 9, we successfully immunized our patient with live‐attenuated MR, varicella, and mumps vaccines without any side effects. Subsequently, we administered VZV and mumps booster vaccines and obtained sufficient titers.

As described above, canakinumab appears to be effective and safe in infants with NOMID/CINCA syndrome. In the present case, we started treatment early to avoid irreversible sequelae. Moreover, we successfully immunized the patient with live‐attenuated vaccines concomitantly with canakinumab treatment. Thus, a normal vaccination schedule may be possible in these patients to avoid acquiring vaccine‐preventable diseases. Accordingly, further studies of live‐attenuated vaccines in these patients are urgently needed to improve their quality of life.

## Authorship

MW: cared for the patient and drafted most of the manuscript. RN: helped with the diagnosis, advised on therapy, and was involved in drafting and revising the manuscript. YF: cared for the patient. TH: helped with the diagnosis and advised on therapy. AO and TS: were the senior physicians responsible for the care of the patient and revision of the manuscript.

## Conflict of Interest

All authors have no conflict of interests to disclose.
